# Distributed Water Pollution Source Localization with Mobile UV-Visible Spectrometer Probes in Wireless Sensor Networks

**DOI:** 10.3390/s18020606

**Published:** 2018-02-16

**Authors:** Junjie Ma, Fansheng Meng, Yuexi Zhou, Yeyao Wang, Ping Shi

**Affiliations:** 1 Chinese Research Academy of Environmental Sciences, Beijing 100012, China; majunjie85@gmail.com (J.M.); mengfs@craes.org.cn (F.M.); zhouyuexi@263.net (Y.Z.); 2 China National Environmental Monitoring Centre, Beijing 100012, China; 3 Yiwen Environmental Science Technology Co., Ltd., Guangzhou 510663, China; shiping@yiwenkeji.com

**Keywords:** pollution source localization, wireless sensor networks, mobile nodes, UV-visible spectroscopy, water quality multi-parameter, distributed algorithm, particle swarm optimization

## Abstract

Pollution accidents that occur in surface waters, especially in drinking water source areas, greatly threaten the urban water supply system. During water pollution source localization, there are complicated pollutant spreading conditions and pollutant concentrations vary in a wide range. This paper provides a scalable total solution, investigating a distributed localization method in wireless sensor networks equipped with mobile ultraviolet-visible (UV-visible) spectrometer probes. A wireless sensor network is defined for water quality monitoring, where unmanned surface vehicles and buoys serve as mobile and stationary nodes, respectively. Both types of nodes carry UV-visible spectrometer probes to acquire in-situ multiple water quality parameter measurements, in which a self-adaptive optical path mechanism is designed to flexibly adjust the measurement range. A novel distributed algorithm, called Dual-PSO, is proposed to search for the water pollution source, where one particle swarm optimization (PSO) procedure computes the water quality multi-parameter measurements on each node, utilizing UV-visible absorption spectra, and another one finds the global solution of the pollution source position, regarding mobile nodes as particles. Besides, this algorithm uses entropy to dynamically recognize the most sensitive parameter during searching. Experimental results demonstrate that online multi-parameter monitoring of a drinking water source area with a wide dynamic range is achieved by this wireless sensor network and water pollution sources are localized efficiently with low-cost mobile node paths.

## 1. Introduction

Water quality monitoring is an important foundation for water environmental protection and water resource management which have become major issues receiving much concern in social sustainable development. Once pollution accidents, such as chemical leaks, occur in surface waters, especially in drinking water source areas, the safety of urban water supply will be endangered [1]. Efficient identification and localization of pollution sources should be performed in water quality monitoring systems, facilitating timely and accurate emergency treatment and reducing harmful influences. The qualitative and quantitative determinations of pollutants are often made by chemical or chromatographic analysis in traditional water quality monitoring systems [2]. Nevertheless, because of some drawbacks, such as complex sample pretreatment course, long measurement period, and the requirement of chemical reagents, these systems are not suitable for applications facing sudden pollution accidents. UV-visible spectroscopy has been attracting growing attention in this application field with its advantages of fast response, in-situ multi-parameter analysis, no secondary pollution, and low maintenance costs [3,4,5]. Existing studies on water quality monitoring with UV-visible absorption spectra include building analytical models for certain parameters, such as total organic carbon (TOC) and chemical oxygen demand (COD) [6,7,8], correcting turbidity influences [9], denoising spectral data [10,11], and developing spectral compression approaches [12]. However, few efforts have been made to construct one synthesis analytical model of multiple parameters, considering the inherent features of continuous spectra. Moreover, the sensing requirement of UV-visible spectrometer probes in the scenario of drinking water source areas facing pollution accidents has not been well discussed. For each UV-visible spectrometer probe, the potential pollutant concentration of sensing object varies in an extremely wide range during water quality monitoring. With a fixed optical path, a common UV-visible spectrometer has a fixed measurement range and cannot provide optimal performance at both high and low pollutant concentrations, leading to loss of accuracy or even invalid results in the extended range. More importantly, since there are complicated pollutant spreading conditions, including pollutant concentration, wind direction, wind velocity, water flow direction, water flow velocity, etc., not only stationary UV-visible spectrometer probes but also mobile ones should be involved in the water quality monitoring. Wireless sensor networks (WSNs) can implement various complicated tasks in the sensing field via a number of smart wireless sensor nodes with sensing, storage, processing and communication capabilities [13,14]. WSNs composed of stationary and mobile UV-visible spectrometer probes have good potential in this application domain, which have not been well explored yet. Therefore, it is significant to investigate a total solution of WSNs to identify and localize water pollution sources efficiently, where the stationary and mobile UV-visible spectrometer probes should be designed to work collaboratively for multi-parameter monitoring in a wide measurement range.

During water pollution source localization, there are complicated pollutant spreading conditions and pollutant concentrations vary in a wide range. This paper constructs a scalable framework to solve these problems and a WSN-based distributed water pollution source localization method is proposed. UV-visible spectrometer probes are well designed for in-situ measurement of multiple parameters, including TOC, nitrate nitrogen, turbidity, etc. An adaptive optical path mechanism is adopted to adjust the measurement range automatically in order to satisfy the performance demands at variable pollutant concentrations. In the established WSNs for water quality monitoring, unmanned surface vehicles (USVs) equipped with the probes act as mobile nodes, while buoys equipped with the probes act as stationary nodes. A novel distributed algorithm, Dual-PSO, is presented to solve the problems in pollution source localization, providing a scalable solution for different applications. One particle swarm optimization (PSO) procedure computes the water quality multi-parameter measurements on each node, utilizing UV-visible absorption spectra, while the other one finds the global solution of the pollution source position, regarding mobile nodes as particles. Meanwhile, the entropy of water quality multi-parameter distribution is introduced as a metric to dynamically recognize the most sensitive parameter of pollution sources during searching. In the experiments of water quality monitoring, the efficiency of quantitative multi-parameter analysis and pollution source localization is verified.

Our former studies were mainly on quantification in total-reflection X-ray fluorescence analysis and target tracking in WSNs. This research focuses on the distributed water pollution source localization method in WSNs. Experience of spectral analysis and network deployment is gained in the former research [15]. However, new application scenarios of WSNs and the very purpose for water pollution source localization are particularly discussed here. The rest of this paper is organized as follows: Section 2 presents the establishment of WSNs for water quality monitoring, including configuration and deployment of wireless sensor nodes. In Section 3, the distributed water pollution source localization method is described in detail. The problems during identification and localization of water pollution sources are formulated, and accordingly the Dual-PSO algorithm is given. The experimental results are presented in Section 4, where the distributed water pollution source localization method is performed in the established WSN and its efficiency is evaluated. We conclude the paper in Section 5.

## 2. WSNs for Water Quality Monitoring

In the scenario of drinking water source areas facing pollution accidents, UV-visible spectrometer probes are employed to analyze water quality in WSNs. Since the pollutant concentrations at each probe can vary in a wide range, an adaptive optical path mechanism is developed to guarantee the sensing performance at different concentrations, making the whole water pollution source localization application feasible. Then, wireless sensor nodes are defined, where buoys and USVs equipped with UV-visible spectrometer probes serve as stationary and mobile nodes respectively. Besides, the deployment scheme of wireless sensor nodes in WSNs is designed for water quality monitoring.

### 2.1. UV-Visible Spectrometer Probes with Adaptive Optical Path

UV-visible Spectroscopy has advantages of fast response, in-situ multi-parameter analysis, no secondary pollution, and low maintenance costs, receiving for these reasons widespread attention, especially in the field of surface water quality monitoring. For traditional UV-visible spectrometer probes, the fixed optical path may lead to loss of accuracy or even invalid results at high or low pollutant concentrations, due to the instrumental factors and the sample properties. Also, the traditional dilution approach makes the systems more complex and cannot achieve in-situ analysis, which is not suitable for our application. However, reliable sensing at pollutant concentrations varying in a wide range is essential here. Thus, the UV-visible spectrometer probes are not only designed to analyze multiple parameters, including TOC, nitrate nitrogen, turbidity, etc., but also improved to adjust the optical path dynamically with an adaptive optical path mechanism.

**Figure 1:**
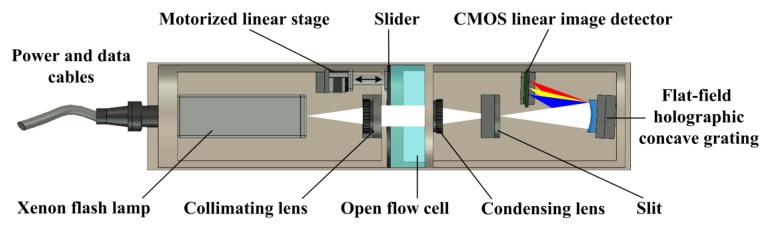
The structure of the UV-visible spectrometer probe with adaptive optical path.

**Figure 2:**
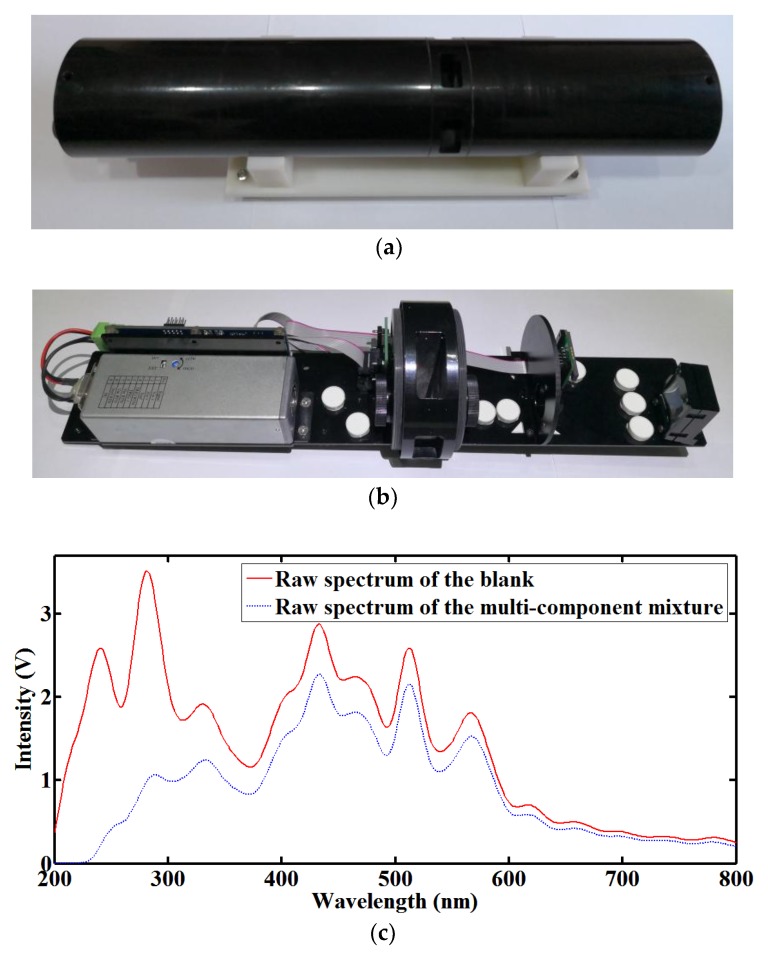
The prototype of the UV-visible spectrometer probe: (**a**) The outside view; (**b**) The internal structure; (**c**) The typical raw spectra of the blank and the multi-component mixture.

The structure of an UV-visible spectrometer probe with an adaptive optical path is shown in Figure 1. With compact design, it mainly comprises a xenon flash lamp, a collimating lens, a condensing lens, a slit, a flat-field holographic concave grating, a complementary metal oxide semiconductor (CMOS) linear image detector, a motorized linear stage and a slider. The xenon flash lamp light source, which delivers high stability and long service life, produces a broad spectral output from 185 nm to 2000 nm with a rated power of 5 W. The flat-field holographic concave grating works between 200 nm and 800 nm with reduced aberrations, bringing benefits of low light losses and simplified optical system. The CMOS linear image detector has 512 pixels, a spectral response range from 200 nm to 1000 nm, and on-chip charge amplifiers. During the operation of the UV-visible spectrometer probe, the light from the xenon flash lamp is collimated by the collimating lens and the beam passes through the water in the open flow cell. Then, the beam after absorption is condensed by the condensing lens and emitted from the slit. Finally, the flat-field holographic concave grating acts as a spectroscopic element and also an imaging element, while the CMOS linear image detector records the UV-visible absorption spectra. The UV-visible absorption spectra has an effective wavelength range from 200 nm to 800 nm and a resolution of 3 nm. Especially, the slider is driven by the motorized linear stage with a maximum speed of 10 mm/s, which makes the optical path in the open flow cell adjustable from 2 mm to 30 mm. The power and data cable, which supports power supply and data transmission, as well as the probe encapsulation are waterproof, so the whole probe can be placed in the water for in-situ analysis, the UV-visible absorption spectra can be acquired by a processor module for further process, and the optical path can be adaptive to internal and external information. As shown in Figure 2, the prototype of the UV-visible spectrometer probe is developed, which is used to analyze multiple parameters. Figure 2a shows the outside view of the prototype, Figure 2b shows the internal structure of the prototype, and Figure 2c shows the typical raw spectra of the blank and the multi-component mixture with TOC at 16 mg/L, nitrate nitrogen at 8 mg/L, nitrite nitrogen at 2 mg/L and turbidity at 20 NTU.

According to the Beer-Lambert law, the absorbance of a monochromatic beam which passes through a homogeneous medium is proportional to the product of the absorbing layer thickness and the absorbing component concentration [16,17]. For the absorbing component *i* and the wavelength *λ*, the absorbance *A_λ,i_* can be written as:

(1) $$A_{\lambda ,i}=\log\frac{I_{\lambda ,i}^{0}}{I_{\lambda ,i}^{t}}=K_{\lambda ,i}bC_{i}$$

where $I_{\lambda ,i}^{0}$ is the intensity of the incident radiation, $I_{\lambda ,i}^{t}$ is the intensity of the transmitted radiation, $K_{\lambda ,i}$ is a constant absorption coefficient at a given temperature for a specific medium, *b* is the absorbing layer thickness known as the adjustable optical path in the open flow cell here, and *C_i_* is the absorbing component concentration. For a multi-component mixture tested by this UV-visible spectrometer probe, the total absorbance *A_λ_* of the wavelength *λ* can be calculated as:

(2) $$A_{\lambda }=\log\frac{I_{\lambda }^{0}}{I_{\lambda }^{t}}=\sum_{i=1}^{n}A_{\lambda ,i}=\sum_{i=1}^{n}K_{\lambda ,i}bC_{i}\;\lambda \in [\lambda_{\min},\lambda_{\max}]$$

where $I_{\lambda }^{0}$ and $I_{\lambda }^{t}$ can derive from the UV-visible absorption spectra of a blank and the mixture respectively, *n* is the total number of absorbing components, *λ*_min_ is the minimum wavelength which is 200 nm, and *λ*_max_ is the maximum wavelength which is 800 nm. According to Equation (2), our UV-visible spectrometer probes can adjust the optical path *b* to tune the current absorbance curve at different pollutant concentrations, so that deviations from the Beer-Lambert law is reduced and accurate measurement in a wide dynamic range is feasible. Besides, the optical path adjustment is performed in the in-situ analysis mode and its granularity can be customized. The optical path adjusting rules and the quantitative multi-parameter analysis approach will be stated in Section 3.

### 2.2. Stationary and Mobile Wireless Sensor Nodes

In the sensing field of surface water, smart wireless sensor nodes are expected to complete sensing, storage, processing and communication in order to gather sufficient information of pollutant concentration distribution. Considering the complicated pollutant spreading conditions, not only stationary nodes but also mobile nodes are designed to localize the pollution source. By integration and miniaturization, buoys and USVs serve as stationary and mobile nodes respectively.

**Figure 3:**
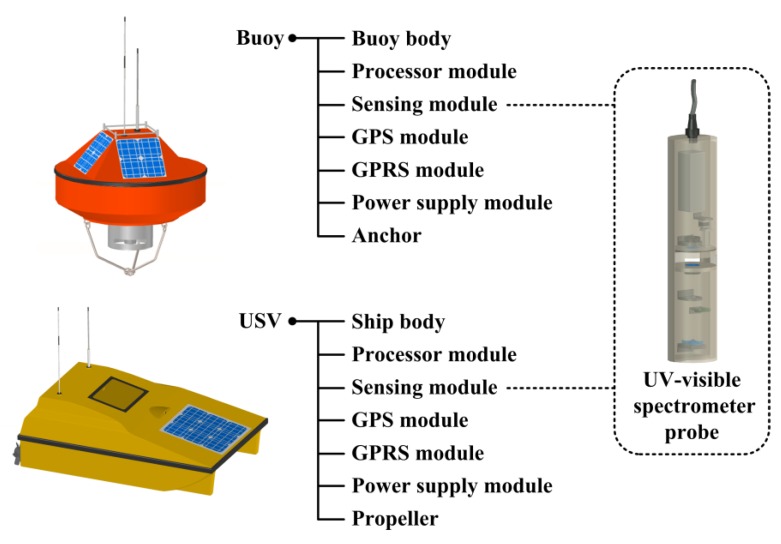
Configuration of stationary and mobile wireless sensor nodes.

As shown in Figure 3, both stationary and mobile nodes are embedded with a processor module, a sensing module, a global positioning system (GPS) module, a general packet radio service (GPRS) module, and a power supply module. The processor module manages the resources of the other modules. The sensing module includes the UV-visible spectrometer probe, cameras and other sensors. The GPS module reports the current position of the node. The GPRS module supports wireless communication between nodes. The power supply module uses lithium batteries with auxiliary power supply from solar panels. In addition, each buoy is fixed by an anchor to prevent floating, while each USV is driven by double propellers with a maximum speed of 3 m/s and a cruise duration of 8 h. In the routine operation of wireless sensor nodes, the processor module acquires UV-visible absorption spectra and other raw data from the sensing module which are used to compute the local water quality multi-parameter measurements, and meanwhile obtains its own current position from the GPS module. Utilizing the GPRS module, the processor module share its information of water quality and node position with other nodes. Also, the processor module may send orders to adjust the optical path of the UV-visible spectrometer probe. For the USVs, there are four navigation modes, including remote control mode, fixed-path cruise mode, autonomous cruise mode and collaborative cruise mode. In the remote control mode, users can control the direction and speed with remote interfaces. In the fixed-path cruise mode, USVs move along predetermined navigation paths. In the autonomous cruise mode, USVs schedule their navigation paths automatically with local information. In the collaborative cruise mode, a group of USVs, usually accompanied by buoys, schedule their navigation paths collaboratively with shared information. USVs and buoys have opportunity to accomplish complicated tasks, such as water pollution source localization, in the collaborative cruise mode, which is mainly discussed in this paper.

### 2.3. Deployment for Water Quality Monitoring

In the water quality monitoring application, WSNs are composed of a number of wireless sensor nodes which are deployed in a two-dimensional sensing field and one sink node which is usually located on the shore. Reasonable deployment of wireless sensor nodes is required to guarantee the basic performance of WSNs.

**Figure 4:**
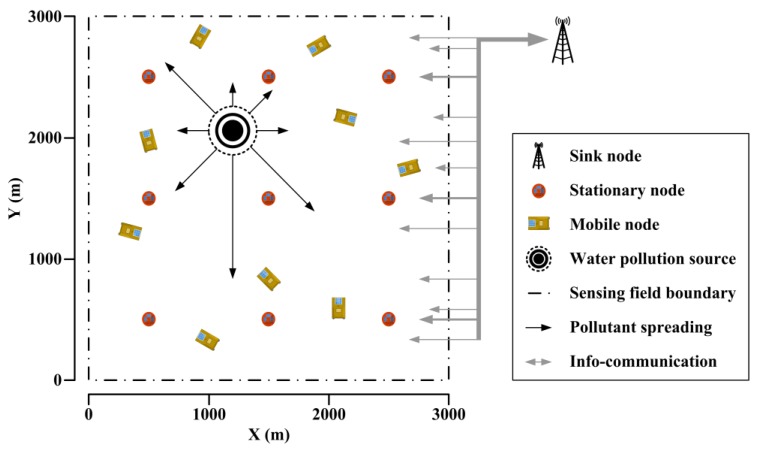
The WSN deployment for water quality monitoring.

As shown in Figure 4, the stationary nodes are deployed regularly in fixed positions to provide stable and uniform coverage, while the mobile nodes are drifting with the waves in random positions to save energy. The sink node gathers local information from stationary nodes and mobile nodes after distributed processing, monitoring the water quality distribution in the sensing field. The task of water pollution source localization is triggered by events. For instance, when the spatial-temporal distribution of water quality satisfies pollution warning conditions or related commands are received from external systems, the water pollution source localization begins. Once a certain kind of pollution accident happens, pollutants spread from the water pollution source. 

Since there are complicated pollutant spreading conditions, including pollutant concentration, wind direction, wind velocity, water flow direction, water flow velocity, etc., especially for large areas of water, it is difficult to build a precise pollutant spreading model from possible water pollution source to wireless sensor nodes in practical applications. Thus, the mobility of wireless sensor nodes is useful to localize the water pollution source. In order to find the water pollution source precisely and quickly, the sink node schedules paths of the mobile nodes based on the complete information of all the nodes. Therefore, the mobile nodes can move to proper positions and report detailed water quality distribution. The water pollution source localization method will be stated in Section 3. Because the sink node maintains a list of wireless sensor nodes to support their joining and leaving, the WSNs are robust and scalable.

## 3. Distributed Water Pollution Source Localization Method

With the defined WSNs, the problems of multi-parameter quantification, pollutant recognition, water pollution source searching and optical path adjustment can be formulated, and then the distributed Dual-PSO algorithm will be proposed to solve these problems and localize the water pollution source.

### 3.1. Problem Formulation

Each wireless sensor node has UV-visible absorption spectra and other raw data from the sensing module as well as its own current position from the GPS module. To localize the water pollution source in WSNs, there are four main problems, including multi-parameter quantification, pollutant recognition, water pollution source searching, and optical path adjustment.

**Figure 5:**
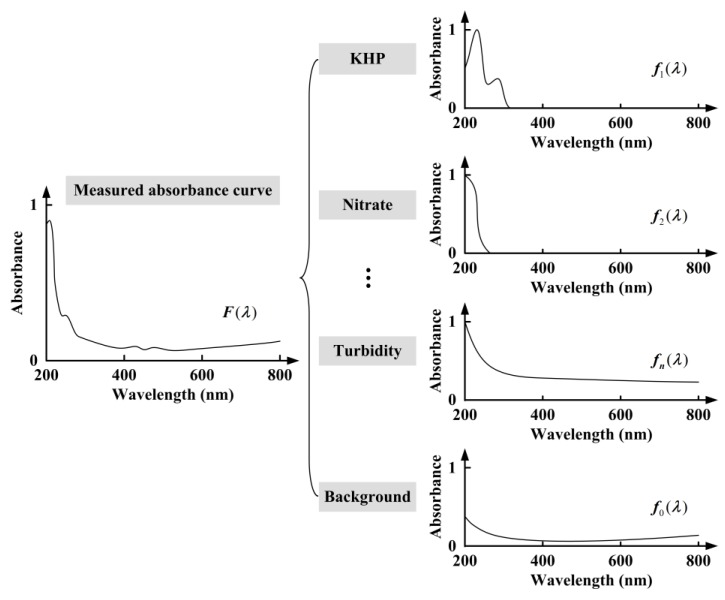
The decomposition strategy of the measured absorbance curve.

First of all, the local water quality multi-parameter measurements should be computed, using the UV-visible absorption spectra. As shown in Equation (2), the Beer-Lambert law is valid for mixtures of absorbing components fulfilling the condition that there are no interactions between these components. The relationship between absorbance curves and multiple parameters, such as TOC, nitrate nitrogen, turbidity, etc., should be investigated. Existing studies on water quality monitoring with UV-visible absorption spectra mostly concentrate on building analytical models for TOC or COD. For example, the regressive model between absorbance curves and TOC is built employing least squares support vector machine (LSSVM) [18]. It has been demonstrated that LSSVM has better performance than other approaches, such as principal component analysis (PCA) and partial least square (PLS). However, few efforts have been made to construct one synthesis analytical model of multiple parameters, considering the inherent features of continuous spectra. A scalable UV-visible spectral decomposition strategy is considered here, based on our former work on total-reflection X-ray fluorescence analysis [15]. As shown in Figure 5, the measured absorbance curve is decomposed into one background curve and a number of characteristic curves of possible components, which can be written as:

(3) $$F(\lambda )=f_{0}(\lambda )+\sum_{i=1}^{n}w_{i}f_{i}(\lambda )\;\lambda \in [\lambda_{\min},\lambda_{\max}]$$

where *F* is the measured absorbance curve, *f*_0_ is the background curve, *f_i_* is the normalized characteristic curve of component *i*, and *w_i_* is the proportionality coefficient of component *i*. The background curve represents the contribution of uncontaminated water, which can be defined as the average absorbance during normal water quality monitoring, since the exogenous pollution accidents are mainly concerned. The normalized characteristic curves of multiple components, such as potassium hydrogen phthalate (KHP), nitrate, turbidity, etc., are shown in the figure, where KHP solution is prepared as a standard sample for TOC testing. According to Equation (2), the normalized characteristic curves can be stated as:

(4) $$f_{i}(\lambda )=\frac{K_{\lambda ,i}}{\underset{\lambda }{\mathrm{MAX}}(K_{\lambda ,i})}\;\lambda \in [\lambda_{\min},\lambda_{\max}]$$

It can be found that the normalized characteristic curves do not depend on the adjustable optical path. These characteristic curves contains corresponding characteristic peaks, while water quality multi-parameter measurements can be derived from the proportionality coefficients. Hence, this problem becomes finding the optimal combination of proportionality coefficients to fit the measured absorbance curve. The objective function of combinatorial optimization problem can be formulated as:

(5) $$O(w_{1},w_{2},\ldots ,w_{n})=\int_{\lambda_{\min}}^{\lambda_{\max}}{\left[F(\lambda )-f_{0}(\lambda )-\sum_{i=1}^{n}w_{i}f_{i}(\lambda )\right]}^{2}d\lambda$$

By minimizing the objective function, the optimal set of proportionality coefficients can be found, where intelligent computing can be applied to solve the combinatorial optimization problem [19,20,21,22,23,24,25,26,27]. According to Equations (2)–(4), the water quality multi-parameter measurements can be calculated as:

(6) $$C_{i}=\frac{w_{i}}{b\underset{\lambda }{\mathrm{MAX}}(K_{\lambda ,i})}\;\lambda \in [\lambda_{\min},\lambda_{\max}]$$

The constant absorption coefficient *K_λ,i_* can be determined by calibration, and the optical path *b* is known during testing. This strategy is suitable for multi-parameter quantification in various applications, because the characteristic curves of possible components can be added into the framework flexibly. Benefitting from optimal fitting in the whole spectral scope and the usage of inherent characteristic curves, it has potential to obtain high accuracy.

Then, the most sensitive parameter which reflects the water pollution source best should be recognized among the multiple parameters. In traditional water quality monitoring systems, the parameter of which the data exceed an established threshold is usually regarded as the most sensitive parameter. However, the establishment of threshold is based on experience, and it may lead to false positive alarms. The water quality distribution should be used to recognize the most sensitive parameter in order to avoid such problems. While the mobile nodes move to more positions in our case, more detailed water quality distribution can be obtained. Assuming that the number of stationary nodes and mobile nodes is *s* and *m* respectively, water quality multi-parameter measurements on the stationary nodes can be written as:

(7) $$S^{\left(j\right)}(x,y)=[C_{1}^{\left(j\right)},C_{2}^{\left(j\right)},\ldots ,C_{n}^{\left(j\right)}]\;j=1,2,\ldots ,s$$

and those on the mobile nodes can be written as:

(8) $$M^{\left(k,l\right)}(x,y)=[C_{1}^{\left(k,l\right)},C_{2}^{\left(k,l\right)},\ldots ,C_{n}^{\left(k,l\right)}]\;k=1,2,\ldots ,m$$

where *x* and *y* are X position and Y position of the wireless sensor nodes in the sensing field respectively, and *l* indicates the movement times of mobile nodes. The distribution of parameter *i* can be described as:

(9) $$D_{i}(x,y)=\{\begin{array}{cc} C_{i}^{\left(j\right)} & (x,y)\;\mathrm{is}\;\mathrm{the}\;\mathrm{positon}\;\mathrm{of}\;\mathrm{stationary}\;\mathrm{node}\;j\\ C_{i}^{\left(k,l\right)} & (x,y)\;\mathrm{is}\;\mathrm{the}\;l-\mathrm{th}\;\mathrm{position}\;\mathrm{of}\;\mathrm{mobile}\;\mathrm{node}\;k \end{array}$$

Thus, pattern recognition should be performed to distinguish a certain distribution from the others in order to find the most sensitive parameter.

Moreover, the mobility of wireless sensor nodes is used to search for the water pollution source. In this paper, it is assumed that the position of water pollution source is fixed, the pollutant concentration at this position is the highest, and the water quality distribution keep stable during searching. Since the sink node gathers the information of all the nodes, it can schedule paths of the mobile nodes utilizing the distribution of the most sensitive parameter. Hence, it becomes an extremum seeking problem in the sensing field. Also, intelligent computing can be considered to solve this problem.

In addition, the sensing object of wireless sensor nodes varies from high-quality surface water to heavily polluted water, so the optical path of each wireless sensor node should be properly adjusted to achieve reliable sensing. According to Equation (2), the absorbance curve can be tuned at different pollutant concentrations with customized granularity. Assuming that the optical path can be adjusted exponentially, it can be written as:

(10) $$b=b_{0}\varphi^{d}\;b\in [b_{\min},b_{\max}]$$

where *b*_0_ is the default optical path, *ϕ* is the scaling unit, *d* is the scaling coefficient which is an integer, *b*_min_ is the minimum optical path which is 2 mm, and *b*_max_ is the maximum optical path which is 30 mm. Commonly, the same absorption coefficient *K_λ,i_* can be simply used when the optical path is adjusted, so Equation (2) can be stated as:

(11) $$A_{\lambda }=A_{\lambda }^{0}\varphi^{d}\;\lambda \in [\lambda_{\min},\lambda_{\max}]$$

where:

(12) $$A_{\lambda }^{0}=\sum_{i=1}^{n}K_{\lambda ,i}b_{0}C_{i}\;\lambda \in [\lambda_{\min},\lambda_{\max}]$$

Therefore, the absorbance curve of the adjusted optical path can be predicted with Equation (11), which can be utilized to set the optical path adjusting rules.

### 3.2. Dual-PSO Algorithm

**Figure 6:**
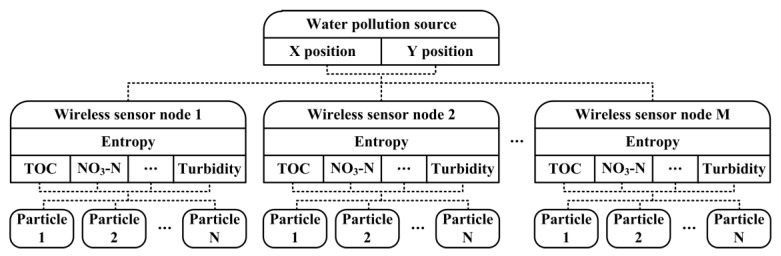
The diagram of the Dual-PSO algorithm.

Considering the mentioned requirements of water pollution source localization and the capacities of WSNs, a distributed algorithm is developed here. For the problems of multi-parameter quantification and water pollution source searching, similar intelligent computing approaches are considered. Some algorithms, such as steepest descent and genetic algorithms (GA), may be discussed in optimization problems [28]. It has been shown that GA has better performance than steepest descent. In our case, however, the mobile nodes act as natural particles in water pollution source searching, different solution dimensions of proportionality coefficients require different granularity in multi-parameter quantification, and the whole procedure needs to be quick and low cost, where particle swarm optimization (PSO) is preferred. Kennedy et al., developed PSO in 1995 based on the analogy of swarms of birds and fish schools [29,30]. PSO is an efficient optimization tool for solving combinatorial optimization problems and dynamic optimization problems. Like other evolutionary algorithms, PSO uses fitness as criterion to evolve the behavior of the solution population. Potential solutions, namely particles, fly through the searching space. Each particle keeps track of the best position it has achieved so far, which represents a particle experiment. Another kind of experiment is the best position which has been achieved by any companion of the particle so far. The particle velocity is constantly adjusted according to the two kinds of experiences. The diagram of the Dual-PSO algorithm is shown in Figure 6, where one PSO procedure computes the water quality multi-parameter measurements on each wireless sensor node and the other one searches for the water pollution source with real particles. With distributed processing, only a small amount of necessary information, such as multi-parameter measurements and position coordinates, is exchanged between nodes, otherwise centralized processing demands more communication cost for raw data transmission. Meanwhile, the entropy of water quality multi-parameter distribution which reflects the disorder degree is introduced as a metric to dynamically recognize the most sensitive parameter during searching, because it has low computational complexity and does not need high resolution input [31,32,33]. Besides, the optical path adjusting rules are set to work in a proper absorbance range adaptively for each wireless sensor node so that reliable sensing at different pollutant concentrations is guaranteed. The pseudo-code for the Dual-PSO algorithm is outlined in Algorithm 1, where more water quality parameters from UV-visible spectrometer probes or other sensors, more wireless sensor nodes and larger sensing fields can be easily extended.

**Algorithm 1:** Dual-PSOOne PSO procedure is performed globally to search for the water pollution source on the sink node and wireless sensor nodes. It is assumed that there are *M* wireless sensor nodes, of which *s* are stationary nodes and *m* are mobile nodes. For α=1,2,…,M  $X_{\alpha }$ represents the current position of wireless sensor node α:
$$X_{\alpha }(1)=[x,y]$$  $U_{\alpha }$ represents the current velocity of wireless sensor node α:
$$U_{\alpha }(1)=[\Delta x,\Delta y]$$  $X_{\alpha }^{G}$ represents the local best position so far, which is initialized as:
$$X_{\alpha }^{G}(1)=X_{\alpha }(1)$$
End The maximum iteration of global PSO is set as $n_{G}$. For $t_{G}=1,2,\ldots ,n_{G}$  For α=1,2,…,M   The absorbance level on wireless sensor node α is defined as:
$${\bar{A}}^{\left(\alpha \right)}=\frac{\int_{\lambda_{1}}^{\lambda_{2}}F^{\left(\alpha \right)}(\lambda )d\lambda }{\lambda_{2}-\lambda_{1}}$$
   where $F^{\left(\alpha \right)}$ is the current absorbance curve. There are intense absorption in the ultraviolet band, so *λ*_1_ and *λ*_2_ are usually set as 200 nm and 300 nm respectively.   Assuming the current optical path is:
$b^{\left(\alpha \right)}=b_{0}\varphi^{d}$   the optical path adjusting rules is set as:
$$b^{\left(\alpha \right)}=\{\begin{array}{cc} b_{0}\varphi^{d+\Delta d} & {\bar{A}}^{\left(\alpha \right)}<{\bar{A}}_{\min}\;\mathrm{and}\;{\bar{A}}_{\min}\varphi^{-\Delta d}\leq {\bar{A}}^{\left(\alpha \right)}<{\bar{A}}_{\min}\varphi^{-\Delta d+1}\\ b_{0}\varphi^{d} & {\bar{A}}_{\min}\leq {\bar{A}}^{\left(\alpha \right)}\leq {\bar{A}}_{\max}\\ b_{0}\varphi^{d-\Delta d} & {\bar{A}}^{\left(\alpha \right)}>{\bar{A}}_{\max}\;\mathrm{and}\;{\bar{A}}_{\max}\varphi^{\Delta d-1}<{\bar{A}}^{\left(\alpha \right)}\leq {\bar{A}}_{\max}\varphi^{\Delta d} \end{array}$$
   where Δd is a positive integer representing the adjustment amount, ${\bar{A}}_{\min}$ is the minimum absorbance level, and ${\bar{A}}_{\max}$ is the maximum absorbance level.    With the adjusted optical path $b^{\left(\alpha \right)}$, the absorbance curve $F^{\left(\alpha \right)}$ is updated.    The other PSO procedure is performed locally to compute water quality multi-parameter measurements on wireless sensor node α and the population of particles is set as *N*.   For β=1,2,…,N       $P_{\beta }$ represents the current solution, initialized randomly in the solution space:
$$P_{\beta }(1)=[w_{1},w_{2},\ldots ,w_{n}]$$       $V_{\beta }$ represents the current velocity, initialized as a random velocity:
$$V_{\beta }(1)=[v_{1},v_{2},\ldots ,v_{n}]$$       $P_{\beta }^{G}$ represents the local best solution so far, which is initialized as:$$P_{\beta }^{G}(1)=P_{\beta }(1)$$   End   The maximum iteration of local PSO is set as $n_{L}$.   For $t_{L}=1,2,\ldots ,n_{L}$       The global best solution $P^{G}$ is defined as:$$O(P^{G}(t_{L}))=\underset{\beta }{\mathrm{MIN}}(O(P_{\beta }^{G}(t_{L})))\;\beta =1,2,\ldots ,N$$       For β=1,2,…,N         The weighted particle velocity is updated as:$$V_{\beta }(t_{L}+1)=\eta (t_{L})V_{\beta }(t_{L})+c_{1}R_{1}[P_{\beta }^{G}(t_{L})-P_{\beta }(t_{L})]+c_{2}R_{2}[P^{G}(t_{L})-P_{\beta }(t_{L})]$$         where *R*_1_ and *R*_2_ are two separate random numbers between 0 and 1, while *c*_1_ and *c*_2_ are acceleration constants which represent the weight of acceleration terms that pull each particle toward the local best solution and the global best solution. Besides, *η* is a inertia weight which decreases during iterations:$$\eta (t_{L})=0.9-\frac{t_{L}}{n_{L}}\times 0.5$$         A large inertia weight facilitates global searching while a small inertia weight facilitates local searching. Hence, particles converge to the neighborhood of global optimal solution smoothly in the prophase and to the global optimal solution quickly in the anaphase.         The solution of each particle is updated as:$$P_{\beta }(t_{L}+1)=P_{\beta }(t_{L})+V_{\beta }(t_{L}+1)$$         The local best solution is updated as:$$P_{\beta }^{G}(t_{L}+1)=\{\begin{array}{cc} P_{\beta }^{G}(t_{L}) & O(P_{\beta }(t_{L}+1))\geq O(P_{\beta }^{G}(t_{L}))\\ P_{\beta }(t_{L}+1) & O(P_{\beta }(t_{L}+1))<O(P_{\beta }^{G}(t_{L})) \end{array}$$       End   End   The optimization result of the proportionality coefficients is recorded as:$$P_{\left(\alpha \right)}^{G}=[w_{1}^{\left(\alpha \right)},w_{2}^{\left(\alpha \right)},\ldots ,w_{n}^{\left(\alpha \right)}]$$   Then the current water quality multi-parameter measurements on wireless sensor node α can be calculated as:$$C_{i}^{\left(\alpha \right)}=\frac{w_{i}^{\left(\alpha \right)}}{b^{\left(\alpha \right)}\underset{\lambda }{\mathrm{MAX}}(K_{\lambda ,i})}\;\lambda \in [\lambda_{\min},\lambda_{\max}]$$  End  A discrete function $D_{i}(x,y)$ of water quality multi-parameter distribution is maintained as:$$D_{i}(x,y)=\{\begin{array}{cc} C_{i}^{\left(j\right)} & \mathrm{Stationary}\;\mathrm{node}\;j\;(j=1,2,\ldots ,s)\;\mathrm{is}\;\mathrm{at}\;(x,y)\\ C_{i}^{\left(k,1\right)} & \mathrm{Mobile}\;\mathrm{node}\;k\;(k=1,2,\ldots ,m)\;\mathrm{is}\;\mathrm{at}\;(x,y)\;\mathrm{in}\;\mathrm{the}\;\mathrm{first}\;\mathrm{iteration}\\ C_{i}^{\left(k,2\right)} & \mathrm{Mobile}\;\mathrm{node}\;k\;(k=1,2,\ldots ,m)\;\mathrm{is}\;\mathrm{at}\;(x,y)\;\mathrm{in}\;\mathrm{the}\;\mathrm{second}\;\mathrm{iteration}\\ \vdots & \vdots \\ C_{i}^{\left(k,t_{G}\right)} & \mathrm{Mobile}\;\mathrm{node}\;k\;(k=1,2,\ldots ,m)\;\mathrm{is}\;\mathrm{at}\;(x,y)\;\mathrm{in}\;\mathrm{the}\;t_{G}-\mathrm{th}\;\mathrm{iteration} \end{array}$$
  where its domain is a set of current and past positions of wireless sensor nodes, and its range is a corresponding set of water quality multi-parameter measurements at these positions.  The entropy of water quality multi-parameter distribution is evaluated.  For i=1,2,…,n   The discrete distribution function of parameter *i* is given. Measurements in its range are sorted in ascending order as:$Q_{1}\leq Q_{2}\leq \ldots \leq Q_{q}\;q=s+mt_{G}$   Normalization is performed as:$0\leq \frac{Q_{2}-Q_{1}}{Q_{q}-Q_{1}}\leq \frac{Q_{3}-Q_{1}}{Q_{q}-Q_{1}}\leq \ldots \leq \frac{Q_{q-1}-Q_{1}}{Q_{q}-Q_{1}}\leq 1\;q=s+mt_{G}$   The entropy of the distribution of parameter *i* is calculated as:$H_{i}=\frac{1}{q}\sum_{\tau =1}^{q}\log\frac{q(Q_{\tau +\delta }-Q_{\tau -\delta })}{\delta (Q_{q}-Q_{1})\rho_{\tau }}\;0<\delta \leq \frac{q}{2}$
   where$$\rho_{\tau }=\{\begin{array}{cc} 1+\frac{\tau -1}{\delta } & 1\leq \tau \leq \delta \\ 2 & \delta <\tau \leq q-\delta \\ 1+\frac{q-\tau }{\delta } & q-\delta <\tau \leq q \end{array}$$
$$\begin{array}{cc} Q_{\tau +\delta }=Q_{q} & \tau >q-\delta \end{array}$$
$$\begin{array}{cc} Q_{\tau -\delta }=Q_{1} & \tau \leq \delta \end{array}$$
  End  The entropies $H_{i}\;(i=1,2,\ldots ,n)$ are compared, and parameter *γ* with the minimum entropy is recognized as the most sensitive parameter of the water pollution source.  The global best position $X^{G}$ is defined as:$$D_{\gamma }(X^{G}(t_{G}))=\underset{\alpha }{\mathrm{MAX}}(D_{\gamma }(X_{\alpha }^{G}(t_{G})))\;\alpha =1,2,\ldots ,M$$  For α=1,2,…,M   The weighted velocity is updated as:$$U_{\alpha }(t_{G}+1)=\eta^{\prime }(t_{G})U_{\alpha }(t_{G})+c_{1}^{\prime }R_{1}^{\prime }[X_{\alpha }^{G}(t_{G})-X_{\alpha }(t_{G})]+c_{2}^{\prime }R_{2}^{\prime }[X^{G}(t_{G})-X_{\alpha }(t_{G})]$$
   where $R_{1}^{\prime }$ and $R_{2}^{\prime }$ are two separate random numbers between 0 and 1, $c_{1}^{\prime }$ and $c_{2}^{\prime }$ are acceleration constants, and $\eta^{\prime }$ is a inertia weight, similarly.   The new position of each wireless sensor node is scheduled as:$$X_{\alpha }(t_{G}+1)=\{\begin{array}{cc} X_{\alpha }(t_{G}) & \mathrm{Wireless}\;\mathrm{sensor}\;\mathrm{node}\;\alpha \;\mathrm{is}\;a\;\mathrm{stationary}\;\mathrm{node}\\ X_{\alpha }(t_{G})+U_{\alpha }(t_{G}+1) & \mathrm{Wireless}\;\mathrm{sensor}\;\mathrm{node}\;\alpha \;\mathrm{is}\;a\;\mathrm{mobile}\;\mathrm{node} \end{array}$$   The local best position is updated as:$$X_{\alpha }^{G}(t_{G}+1)=\{\begin{array}{cc} X_{\alpha }^{G}(t_{G}) & D_{\gamma }(X_{\alpha }(t_{G}+1))\leq D_{\gamma }(X_{\alpha }^{G}(t_{G}))\\ X_{\alpha }(t_{G}+1) & D_{\gamma }(X_{\alpha }(t_{G}+1))>D_{\gamma }(X_{\alpha }^{G}(t_{G})) \end{array}$$  End End The optimization result $X^{G}$ of the water pollution source position is recorded.

## 4. Results and Discussion

A simulation scene is constructed as shown in Figure 4. The sensing field is 3000 m long and 3000 m wide, where nine stationary nodes are deployed regularly in fixed positions and nine mobile nodes are drifting with the waves in random initial positions. During water pollution source localization, the sink node gathers multi-parameter measurements and position coordinates from all wireless sensor nodes, and sends the scheduled paths to the mobile nodes. For the designed UV-visible spectrometer probes, multiple parameters, including TOC, nitrate nitrogen, nitrite nitrogen and turbidity, are considered. In the optical path adjusting rules, the default optical path *b*_0_ is set as 10 mm, the scaling unit *ϕ* is set as 2, the minimum absorbance level ${\bar{A}}_{\min}$ is set as 0.3, and the maximum absorbance level ${\bar{A}}_{\max}$ is set as 0.7.

**Figure 7:**
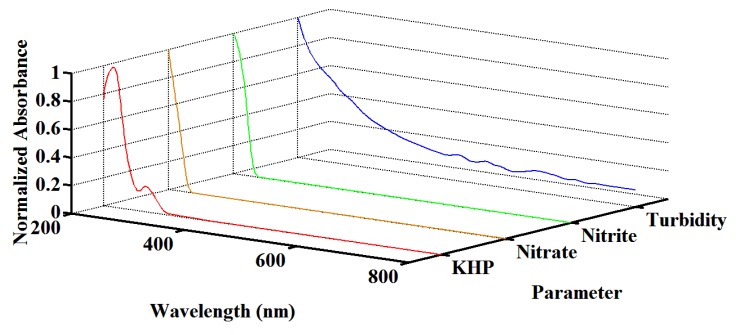
Normalized characteristic curves of multiple parameters.

**Figure 8:**
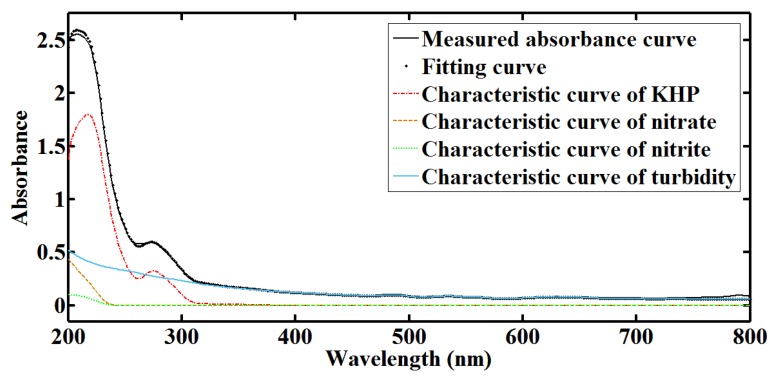
The fitting curve of measured absorbance curve in multi-parameter quantification.

The efficiency of multi-parameter quantification is examined first. KHP solution is prepared as a standard sample for TOC testing, potassium nitrate solution is prepared as a standard sample for nitrate nitrogen testing, sodium nitrite solution is prepared as a standard sample for nitrite nitrogen testing, and formazine solution is prepared as a standard sample for turbidity testing. Both single-parameter samples and multi-parameter samples are used in the experiments. As shown in Figure 7, normalized characteristic curves of multiple parameters are acquired. For a measured absorbance curve, quantification with Dual-PSO is accomplished on a single wireless sensor node. A multi-parameter sample with TOC at 16 mg/L, nitrate nitrogen at 8 mg/L, nitrite nitrogen at 2 mg/L and turbidity at 20 NTU is tested. Figure 8 shows the measured absorbance curve, the fitting curve and the multi-parameter contributions. It can be found that the measured absorbance curve is approximated with high accuracy. More multi-parameter samples are tested to examine the performance utilizing adaptive optical path. Table 1 gives the relative error (RE) and the relative standard deviation (RSD) of TOC, nitrate nitrogen (NO_3_-N) and turbidity measurements, compared with the LSSVM method. The optical path (OP) switches between 5 mm, 10 mm and 20 mm in our method, while it keeps 10 mm in LSSVM. It is shown that the quantification performance is enhanced by our method, especially in the case that there is opportunity to optimize the optical path. With the designed UV-visible spectrometer probe and the Dual-PSO algorithm, optimal fitting in the whole spectral scope is taken into account and deviations from the Beer-Lambert law are reduced, so RE and RSD are smaller and do not vary drastically when the pollutant concentrations vary in a wide range. Besides, simultaneous and fast quantification of multiple parameters is achieved without the requirement of a large number of training populations.

**Figure 9:**
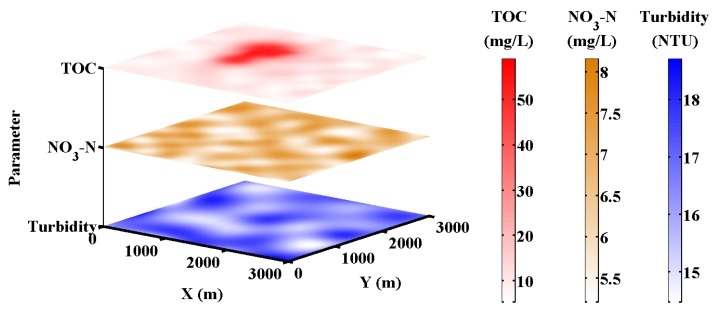
The simulated water quality multi-parameter distribution.

**Figure 10:**
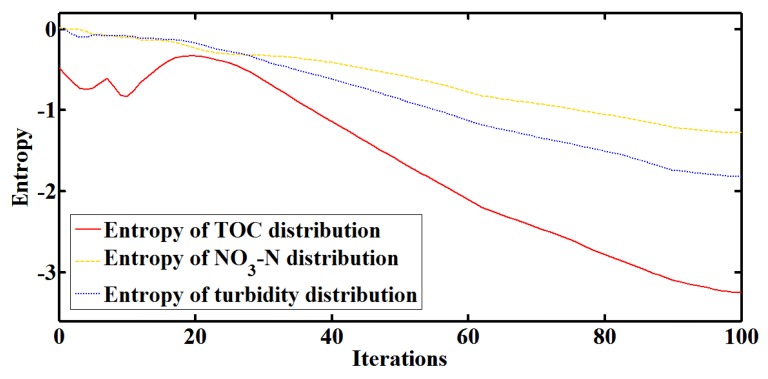
The entropy of water quality multi-parameter distribution during searching.

In the sensing field, the water quality multi-parameter distribution is simulated as shown in Figure 9. Quantitative analysis of TOC, nitrate nitrogen and turbidity are considered, and TOC is set as the most sensitive parameter which reflects the water pollution source best. The Dual-PSO algorithm is performed on the wireless sensor nodes and the sink node to identify and localize the water pollution source. The entropy of water quality multi-parameter distribution during searching is calculated dynamically as shown in Figure 10. It can be found that the entropy of TOC distribution keeps lower than that of nitrate nitrogen or turbidity distribution, and the difference becomes more significant with more detailed distribution during searching. Thus, the entropy is an effective metric to recognize the most sensitive parameter, of which the disorder degree is lower than that of the other parameters which are more evenly distributed. In this way, more parameters from the UV-visible spectrometer probe or other sensors can be easily added into the Dual-PSO algorithm for recognition.

**Figure 11:**
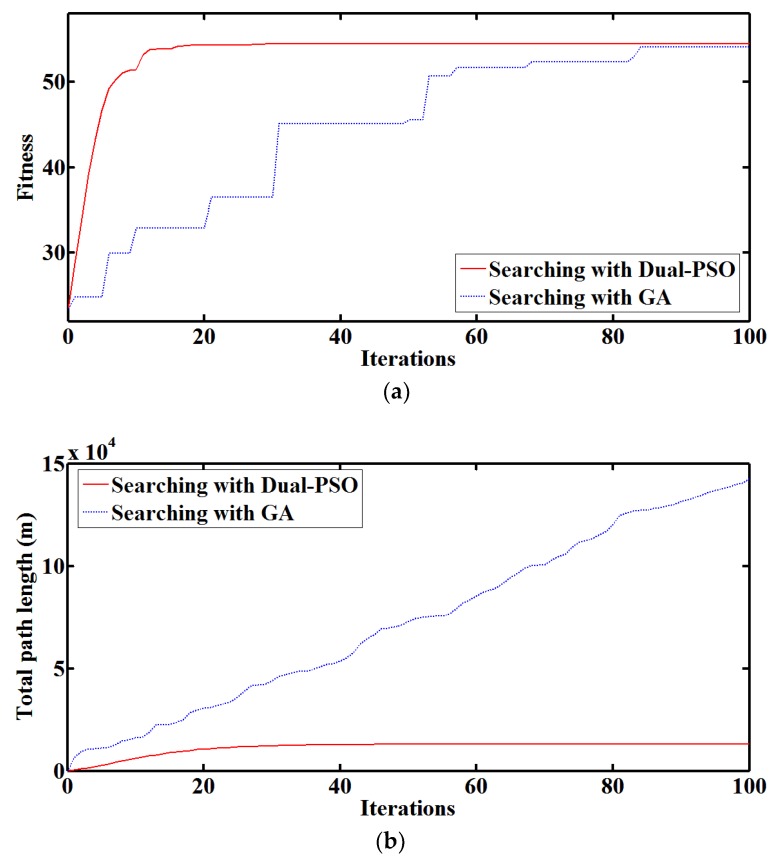
Comparison of water pollution source searching with Dual-PSO and GA: (**a**) The convergence curves; (**b**) The total path length of mobile nodes during searching.

In Figure 11, water pollution source searching with Dual-PSO and GA is compared, where GA uses the same swarm of mobile nodes as Dual-PSO. It can be seen that the convergence of Dual-PSO is faster than that of GA and the total path length of mobile nodes in Dual-PSO is much shorter than that in GA. Dual-PSO finished searching within 20 iterations, while GA does not achieve the same searching result after even 80 iterations. 

The Dual-PSO algorithm enjoys high convergence speed and global optimization ability. In Dual-PSO, the scheduled path of each mobile nodes is smoother, the path length of each mobile node within one single iteration decreases during searching, and the path lengths of different mobile nodes are balanced. That is because PSO is based on the analogy of swarms of birds and fish schools. Given the maximum speed of mobile nodes, each iteration can be completed within 1 minute in Dual-PSO, while the time consumption of each single iteration is unpredictable and the longest iteration in this case lasts over 5 min in GA. Moreover, the step length of searching can be adaptive to different mobile abilities or deployment densities in Dual-PSO. Therefore, efficient water pollution source localization can be achieved by the Dual-PSO algorithm with less time consumption, less power consumption and more feasible navigation paths. Also, it can be realized that water quality measurements, no matter direct measurements or indirect measurements, which reflect the pollutant concentration distribution is applicable for searching, which means that there is opportunity to miniaturize and strengthen the wireless sensor nodes with more optional sensors and soft measurement models. During water pollution source localization, the water quality multi-parameter distribution is simulated under complicated pollutant spreading conditions, and the proposed method can find the water pollution source within 20 min. When pollutant concentrations vary in a wide range, the UV-visible spectrometer probes keep acceptable sensing performance. The Dual-PSO algorithm obtains better multi-parameter quantification results than LSSVM and also obtains better water pollution source searching results than GA. Besides, the most sensitive parameter is efficiently recognized using the entropy of water quality multi-parameter distribution during localization.

## 5. Conclusions

To identify and localize water pollution sources efficiently in surface water-facing pollution accidents, this paper proposes a distributed water pollution source localization method in WSNs. Firstly, WSNs are established for water quality monitoring, where wireless sensor nodes equipped with well-designed UV-visible spectrometer probes are deployed to analyze multiple parameters. Buoys and USVs serve as stationary and mobile nodes respectively, and the optical path of each UV-visible spectrometer probe is adjustable. Then, the Dual-PSO algorithm is presented to solve the problems of multi-parameter quantification, pollutant recognition, water pollution source searching and optical path adjustment. One PSO procedure computes the water quality multi-parameter measurements on each wireless sensor node and the other one searches for the water pollution source with real particles. With distributed processing, only a small amount of necessary information, including multi-parameter measurements, position coordinates and scheduled paths, is exchanged between nodes. The entropy of water quality multi-parameter distribution which reflects the disorder degree is introduced as a metric to dynamically recognize the most sensitive parameter during searching. The optical path adjusting rules are set so that each wireless sensor node can work in a proper absorbance range adaptively for reliable sensing. Finally, experiments and simulations demonstrate the efficiency of quantitative multi-parameter analysis and water pollution source localization. The Dual-PSO algorithm enhances the multi-parameter quantification performance without the requirement of a large number of training populations, and it completes the pollution source searching with less time consumption, less power consumption and more feasible navigation paths. The main contribution of this paper is that the total solution of WSNs is investigated to identify and localize water pollution sources efficiently under complicated pollutant spreading conditions, the novel distributed Dual-PSO algorithm is studied particularly to solve the related problems, the adaptive optical path mechanism reduces deviations from the Beer-Lambert law in a wide measurement range, and the scalable framework is constructed to support the extension of more water quality parameters from UV-visible spectrometer probes or other sensors, more wireless sensor nodes and larger sensing fields. This paper focuses on the application scenario of one certain stationary water pollution source, making use of multiple parameters from UV-visible spectrometer probes. In future research, the distributed water pollution source localization method shall be evolved, considering more application scenarios, such as multiple water pollution sources with different pollutants, mobile water pollution sources, interactions between multi-components, extension of parameters, and energy-aware management of mobile nodes.

## Figures and Tables

**Table 1:** Comparison of multi-parameter quantification with Dual-PSO and LSSVM.

Sample No.	Parameter	Concentration	Dual-PSO			LSSVM		
			OP	RE	RSD	OP	RE	RSD
1	TOC	8 mg/L	20 mm	−4.23%	1.03%	10 mm	−6.97%	2.36%
	NO_3_-N	4 mg/L		4.85%	1.49%		6.13%	3.10%
	Turbidity	10 NTU		3.06%	2.05%		5.50%	3.89%
2	TOC	16 mg/L	10 mm	−3.52%	1.12%		−4.16%	1.25%
	NO_3_-N	8 mg/L		4.59%	1.47%		5.73%	1.62%
	Turbidity	20 NTU		2.70%	2.08%		2.82%	2.19%
3	TOC	32 mg/L	5 mm	−7.36%	1.26%		−12.55%	2.85%
	NO_3_-N	16 mg/L		5.02%	1.51%		15.13%	2.93%
	Turbidity	40 NTU		3.92%	2.16%		6.12%	3.67%
